# Supplementary material for: The Release of Non-Extractable Ferulic Acid from Cereal By-Products by Enzyme-Assisted Hydrolysis for Possible Utilization in Green Synthesis of Silver Nanoparticles
Source: Nanomaterials (Basel). 2022 Sep 2;12(17):3053. doi: 10.3390/nano12173053 (PMC9458256; doi:10.3390/nano12173053)
Supplement: Supplementary file 1 [file nanomaterials-12-03053-s001.zip › nanomaterials-1886938-supplementary.pdf]

Supplementary Materials

The Release of Non-Extractable Ferulic Acid from Cereal By-Products by Enzyme-Assisted Hydrolysis for Possible Utilization in Green Synthesis of Silver Nanoparticles

Vitalijs Radenkovs [1,2,*], Karina Juhnevica-Radenkova [1], Dmitrijs Jakovlevs [2], Peteris Zikmanis [1], Daiga Galina [2,3] and Anda Valdovska [2,3]

[1] Processing and Biochemistry Department, Institute of Horticulture, LV-3701 Dobele, Latvia

[2] Research Laboratory of Biotechnology, Division of Smart Technologies, Latvia University of Life Sciences and Technologies, LV-3004 Jelgava, Latvia

[3] Faculty of Veterinary Medicine, Latvia University of Life Sciences and Technologies, LV-3004 Jelgava, Latvia

* Correspondence: vitalijs.radenkovs@llu.lv; Tel.: +371-2865-0011

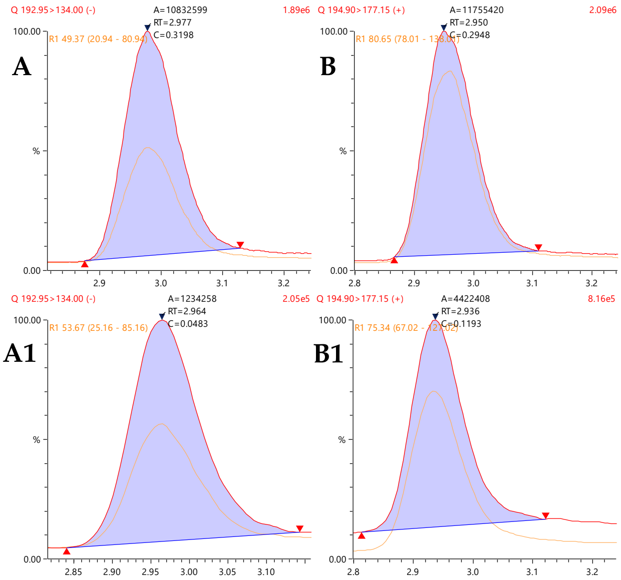

Figure S1. Extracted ion chromatograms (EICs) plotted for *trans*-ferulic acid (A) and *trans*-*iso*-ferulic acid (B) reference standards and for those recovered compounds from bran hydrolysates (A1,B1) following solid-phase extraction with Strata X column using acidified 50% EtOH solution.

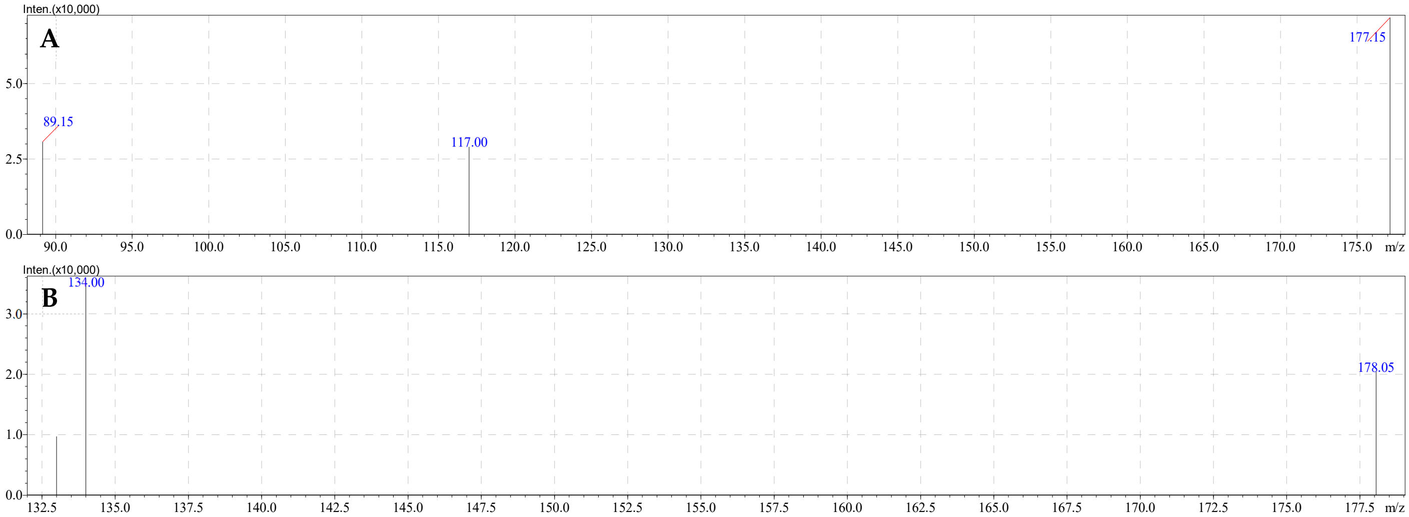

Figure S2. The MRM transitions optimized for selective determination of *trans*-ferulic acid (A) and *trans*-*iso*-ferulic acid (B) using HPLC-ESI-TQ-MS/MS.

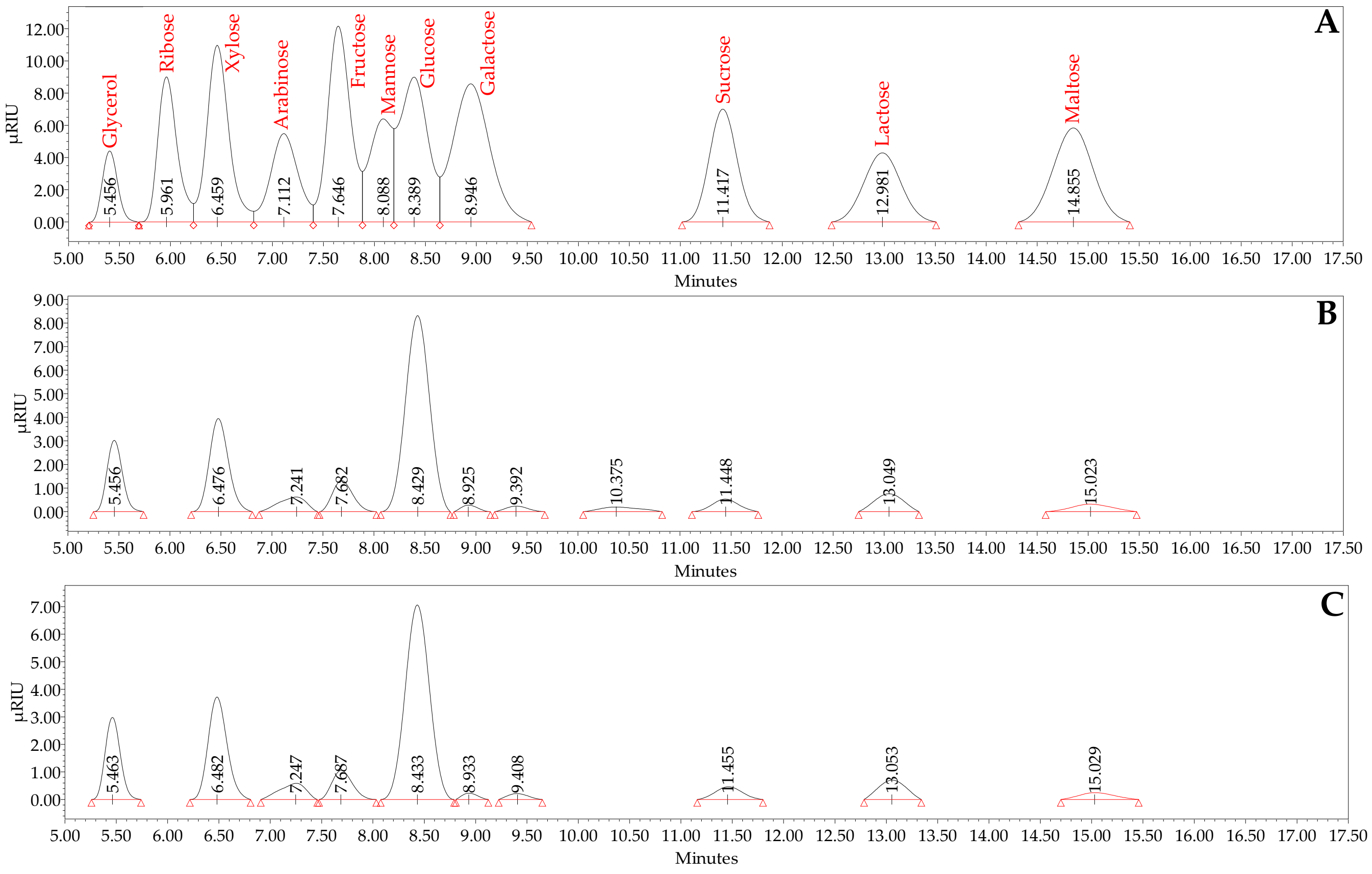

Figure S3. HPLC-RID chromatographic separation of mono- and disaccharides in a mixture of standards (A) rye bran hydrolysate obtained after EH with Viscozyme L and wash-through fraction following solid-phase extraction with Strata X column using acidified 50% EtOH.
